# Paediatric Obsessive-Compulsive Disorder and Depressive Symptoms: Clinical Correlates and CBT Treatment Outcomes

**DOI:** 10.1007/s10802-014-9943-0

**Published:** 2014-10-10

**Authors:** H. M. Brown, K. J. Lester, A. Jassi, I. Heyman, G. Krebs

**Affiliations:** 1 Institute of Psychiatry, King’s College, London, SE5 8AF UK; 2 OCD clinic, Michael Rutter Centre, South London and Maudsley NHS trust, London, SE5 8AZ UK; 3 Institute of Child Health, University College London, London, WC1N 1EH UK

**Keywords:** Obsessive-compulsive disorder, Depression, Correlates, Cognitive-behavioural therapy, Treatment outcomes

## Abstract

Depression frequently co-occurs with paediatric obsessive-compulsive disorder (OCD), yet the clinical correlates and impact of depression on CBT outcomes remain unclear. The prevalence and clinical correlates of depression were examined in a paediatric specialist OCD-clinic sample (*N* = 295; Mean = 15 [7 – 18] years, 42 % female), using both dimensional (Beck Depression Inventory-youth; *n* = 261) and diagnostic (Development and Wellbeing Assessment; *n* = 127) measures of depression. The impact of depressive symptoms and suspected disorders on post-treatment OCD severity was examined in a sub-sample who received CBT, with or without SSRI medication (*N* = 100). Fifty-one per-cent of patients reported moderately or extremely elevated depressive symptoms and 26 % (95 % CI: 18 – 34) met criteria for a suspected depressive disorder. Depressive symptoms and depressive disorders were associated with worse OCD symptom severity and global functioning prior to CBT. Individuals with depression were more likely to be female, have had a psychiatric inpatient admission and less likely to be attending school (*ps* < 0.01). OCD and depressive symptom severity significantly decreased after CBT. Depressive symptoms and depressive disorders predicted worse post-treatment OCD severity (*β*s = 0.19 and 0.26, *p*s < 0.05) but became non-significant when controlling for pre-treatment OCD severity (*β*s = 0.05 and 0.13, *ns*). Depression is common in paediatric OCD and is associated with more severe OCD and poorer functioning. However, depression severity decreases over the course of CBT for OCD and is not independently associated with worse outcomes, supporting the recommendation for treatment as usual in the presence of depressive symptoms.

**Electronic supplementary material:**

The online version of this article (doi:10.1007/s10802-014-9943-0) contains supplementary material, which is available to authorized users.

Obsessive-compulsive disorder (OCD) is a chronic and debilitating condition, affecting around 1 % of young people (Douglass et al. 1995; Flament et al. 1988; Heyman et al. 2001). Paediatric OCD is associated with multiple impairments including those in family and social relationships and school functioning (Piacentini et al. 2003). Cognitive behavioural therapy (CBT) is efficacious in treating paediatric OCD and is associated with a greater effect size than pharmacological treatment (Abramowitz et al. 2006; Watson and Rees 2008). While the majority of young people with OCD improve with CBT, a significant minority fail to show meaningful improvement and an even larger proportion fail to remit (March et al. 2004).

Identifying predictors of poor treatment response is important to inform assessment and treatment strategies. Few predictors of paediatric OCD treatment outcomes have been consistently identified (Garcia et al. 2010; Ginsburg et al. 2008; Storch et al. 2010). In their review, Ginsburg and colleagues concluded that only baseline OCD symptom severity and family dysfunction were consistently associated with poorer response to CBT. Sex, age and age of onset were rarely associated with treatment response. More recently, Garcia and colleagues (2010) demonstrated that diminished insight, greater family accommodation and comorbid psychiatric conditions were associated with poor response to CBT for OCD.

Clinical experience and limited data suggest that psychiatric comorbidity may be associated with poorer outcomes to CBT for a range of psychiatric disorders in youth (Hudson et al. 2013; Ollendick et al. 2008). Comorbid conditions in OCD are common (Tukel et al. 2002). Rates of comorbid depressive disorders are estimated to be around 25 – 40 % in adults (Douglass et al. 1995; Heyman et al. 2001; Overbeek et al. 2002) and 13 – 73 % in children (Ivarsson et al. 2008; Storch et al. 2012), making it one of the most common comorbidities in OCD.

In adults, comorbid depression has been associated with more severe OCD symptoms and specific obsessive-compulsive categories (aggressive, sexual, religious obsessions) in some studies but not others (Besiroglu et al. 2007; Hasler et al. 2005; Hong et al. 2004; Tukel et al. 2006). Comorbid depression has also been variably associated with a range of sociodemographic and clinical features including earlier OCD onset, family history of depression, greater functional impairment and poorer quality of life (Abramowitz et al. 2007; Hong et al. 2004; Huppert et al. 2009).

Only a few studies have examined the correlates of depression in youth with OCD. Concurrent depression has been most consistently associated with greater OCD symptom severity (Canavera et al. 2010; Ivarsson et al. 2008; Storch et al. 2008; Zitterl et al. 2000). In addition, Canavera and colleagues (2010) found that youth with comorbid major depressive disorder (MDD; *n* = 28, aged 10 – 17) demonstrated more social problems and family conflict, and poorer family organisation than age and gender-matched youth with OCD alone. Similarly, Storch et al. (2012) found that both depressive symptoms and depressive disorders were associated with greater OCD symptom severity, but also poorer global functioning in youth with OCD (*n* = 160; aged 7 – 20). Furthermore, functional impairment partially mediated the relationship between OCD and depressive symptom severity, suggesting depression may be a product of OCD and its associated day-to-day impairments; although specific domains of impairment are not well documented.

Theoretically, depression may impede response to CBT for OCD for a number of reasons. Depression is characterised by pervasive hopelessness which may lead to lack of optimism and decrease motivation to engage in treatment. Similarly, reduced insight and low perceived self-competence in youth with depression may limit engagement with therapy (Peris et al. 2010). Alternatively, depression may alter the focus of the therapy; therapists may need to concurrently treat depressive symptoms thus diluting treatment response.

A number of studies have examined the effect of concurrent depressive disorders and/or symptoms on OCD outcomes following CBT. The majority have been conducted with adults and reveal mixed findings. Some have shown that having a comorbid depressive *disorder* is associated with greater post-treatment OCD symptom severity than OCD alone (Abramowitz and Foa 2000; Overbeek et al. 2002). Similarly, adults with extremely elevated depressive *symptoms* showed worse post-treatment OCD symptoms and lower response rates than those with mild or moderately elevated depression (Abramowitz et al. 2000). However, other studies have found no effect of concurrent depressive disorders on CBT outcomes (Abramowitz and Foa 2000; Storch et al. 2010).

Mixed results in adult studies may, at least in part, be attributable to methodological differences. Importantly, only some studies control for pre-treatment differences in OCD symptom severity. For example, whilst Abramowitz and colleagues (2000) found that those with extremely elevated depressive symptoms had more severe OCD following CBT than those with fewer depressive symptoms, they did not examine differences in OCD severity between depression groups prior to treatment. Given associations between depression and OCD severity, it is possible that more severe post-treatment OCD symptoms in depressed adults reflects their greater overall symptom severity compared to non-depressed adults rather than being associated with depression per se. For example, Storch and colleagues (2010) found that whilst adults with OCD and Major Depressive Disorder (MDD) had more severe OCD than those with OCD alone, there was no effect of depression on post-treatment OCD severity when accounting for these baseline differences. Similarly, a study of residential OCD treatment in adults showed that depressive symptoms did not predict post-treatment OCD severity when accounting for pre-treatment OCD severity (Stewart et al. 2006).

Even fewer studies have examined the impact of depression on CBT response in paediatric OCD populations. A small pilot study of group CBT (*N* = 43; aged 7 – 17) found no effect of comorbid MDD on response or remission rates either immediately following CBT or at 6 month follow-up (Farrell et al. 2012). Conversely however, a study of individual CBT (*N* = 96; aged 7 – 19) showed that young people with comorbid MDD, assessed using a structured diagnostic interview, had comparable response rates but lower remission rates than those without MDD following 14 sessions of CBT, suggesting children with OCD and depression do still improve following CBT but are less likely to achieve a full recovery (Storch et al. 2008). However, as with studies with adults, this study did not control for greater pre-treatment OCD severity in children with MDD relative to OCD alone, which would make them less likely to reach the remission cut-off. Meyer and colleagues (2014) examined week-by-week changes in OCD and depressive symptoms across a multimodal (CBT, SSRI, placebo) treatment study of youth (*N* = 56; aged 7 – 17). Multi-level modelling revealed that higher average OCD severity was associated with greater depressive symptoms across treatment but that regardless of initial depressive symptom severity, these symptoms decreased in line with reductions in OCD symptom severity. Finally, a recent study of residential treatment of OCD in adolescents (*N* = 126; aged 13 – 17) showed that depressive symptom severity was not associated with OCD severity at discharge when accounting for differences in OCD severity at admission; instead, depressive symptoms decreased after treatment in line with reductions in OCD (Leonard et al. 2014). Although the largest study to examine this issue, the residential treatment described in this study was multi-modal, including intensive exposure with response prevention (E/RP) in addition to non-E/RP CBT, group work, family sessions and pharmacotherapy. It is unclear to what extent these findings translate to less intensive/holistic treatment approaches.

The current study extends this limited research by exploring the prevalence and clinical correlates of depression and its impact on CBT outcomes in the largest paediatric OCD outpatient sample to date. Unlike previous studies, we examined both depressive symptoms and suspected depressive disorders and compared multiple informants of OCD severity (clinician-, parent- and self-report) in order to clarify previous mixed results. We predicted that depression would be common and associated with more severe OCD symptoms prior to treatment in our sample. We conducted exploratory analyses of possible demographic and clinical correlates including measures of age, gender and functional status. We examined the impact of concurrent depression on outcomes of CBT for OCD. We predicted that depressive symptoms would decrease over the course of CBT. Moreover, we predicted that depression would not be associated with poorer outcome (i.e., more severe obsessive-compulsive symptoms) when controlling for baseline OCD severity.

## Methods

### Participants

All data were collected as part of routine clinical practice and so did not require participant consent. The study was approved by the South London and Maudsley Clinical Audit and Effectiveness Committee. The sample consisted of 295 young people aged 7–18 years (mean = 15 years; 42 % female) consecutively referred to a national and specialist OCD clinic at the Maudsley Hospital, London between 2007 and 2012. All patients met ICD-10 (World Health Organisation 2008) criteria for OCD as confirmed at assessment with the multi-disciplinary team. A subset of 112 young people from the total sample (38 %) received CBT at the clinic. Decisions whether or not to offer CBT were driven by a number of factors including; appropriateness of CBT for the individual, geographical proximity to the clinic, family wishes and funding arrangements. The remaining young people were seen for assessment only and referred back to local Child and Adolescent Mental Health Services (CAMHS). Importantly, those who received treatment at the clinic did not differ significantly in key demographics or obsessive-compulsive or depressive symptom severity from those who did not (see Table 1 in supplementary material). However, those who received treatment at the clinic were more likely to have a first-degree relative with OCD. Almost half the treatment subsample had undertaken previous CBT (48 %) and/or were taking selective serotonin reuptake inhibitors (SSRIs) (43 %). In most cases medication was started and reached a stable dose prior to commencing CBT.

### CBT Treatment

All participants in the treatment subsample received OCD-specific, protocol-driven CBT for young people focusing on exposure with response prevention (E/RP). The CBT protocol involved 14 sessions delivered within 17 weeks, although there was some flexibility. The mean number of sessions was 14 (SD = 5), which were completed over a mean time period of 17 weeks (SD = 10). The first 2 sessions focused on psycho-education about OCD and anxiety; including learning to recognise physical symptoms of anxiety and rate anxiety, and understanding the principle of anxiety habituation. A hierarchy of feared situations is developed in collaboration with the child, and parent where appropriate. From session 3 to 12, E/RP tasks are conducted as guided by the hierarchy with the therapist in sessions and as homework in between sessions. The final 2 sessions focus on relapse prevention. This includes developing a plan for tackling any residual symptoms and identifying an action plan for potential re-emerging symptoms. All CBT sessions were conducted by qualified clinical psychologists or psychiatrists with expertise in child OCD or by trainees under close clinical supervision. Parents were typically included in the initial psychoeducation sessions in order to develop a shared understanding of the problem. Thereafter, parents were involved to varying degrees depending on the child’s developmental level and the extent to which parents were involved in accommodating OCD rituals.

### Measures

#### Standard clinic Information

Demographic and personal measures were collected prior to assessment. These included; age of OCD onset, family history of psychiatric disorders, current medication status, information regarding any previous CBT, history of psychiatric admissions, and current school attendance. Clinician ratings of general functioning were made using the Children’s Global Assessment Scale (CGAS; Shaffer et al. 1983).

#### OCD

Clinician-rated OCD symptoms were assessed before and after CBT using the Children’s Yale-Brown Obsessive Compulsive Scale (CY-BOCS; Scahill et al. 1997); a semi-structured, clinician-rated instrument consisting of a symptom checklist and ratings of OCD-related distress, frequency, interference, resistance and control. Ratings were summed to create total OCD severity scores. The symptom checklist was used to calculate scores for four OCD symptom dimensions; Symmetry, Forbidden thoughts, Cleaning and Hoarding (Bloch et al. 2008). The Children’s Obsessive-Compulsive Inventory (ChOCI; Uher et al. 2008) was used to assess self- and parent-ratings of OCD severity. The ChOCI questionnaire mirrors the CY-BOCS. Both measures demonstrate sound psychometric properties (Scahill et al. 1997; Storch et al. 2004; Uher et al. 2008). Internal consistencies in the current study were high (α > 0.82).

#### Depression

Depressive symptoms were assessed before and after CBT using the Beck’s Depression Inventory- Youth (BDI-Y; Beck et al. 2001); a 20-item self-report questionnaire. Responses were summed and converted to age- and gender-specific t-scores. BDI-Y t-scores were also used to identify children with average or below depression severity (*<*55) and mildly (55 –59), moderately (60 – 69) and severely (≥70) elevated depression. The BDI-Y demonstrates sound psychometric properties (Stapleton et al. 2007). Internal consistency was 0.93 in the current sample.

The Development and Well-being Assessment (DAWBA; Goodman et al. 2000) was used to identify patients meeting criteria for depressive disorders prior to assessment. The DAWBA is a semi-structured interview consisting of closed- and open-ended questions covering a range of emotional and behavioural symptoms. The DAWBA demonstrates substantial agreement with clinician ratings (Cohen’s Kappa (κ = 0.67; Goodman et al. 2000). All families were invited to complete the DAWBA online prior to assessment at the clinic. Computer algorithms of closed responses were used to identify children meeting criteria for a suspected major depressive disorder in line with the DSM-IV (American Psychiatric Association 2000). Complete DAWBA data was available from 127 families at baseline. Families who completed the DAWBA did not differ from non-completers in age, gender, global functioning OCD or depressive symptom severity (see Table 2 in supplementary material). DAWBA data was unavailable post-treatment.

### Analyses

The prevalence of depressive symptom categories and diagnoses were examined using proportions and confidence intervals. The clinical correlates of concurrent depression were examined using Pearson’s correlations for depressive symptoms and *t*-tests and Chi-squared tests for comparing those with and without suspected depressive disorders. Paired *t*-tests were used to identify change in OCD and depressive symptoms severity after treatment. Finally, mixed-model ANOVAs and multiple linear regression models examined whether pre-treatment depression predicted post-treatment OCD severity. Regression analyses controlled for pre-treatment OCD severity, sex and whether or not they were on SSRI mediation. Sample sizes varied for some analyses due to missing data.

## Results

### Attrition Analyses

Participants were included in treatment subsample analyses if they had pre- and post-treatment clinican-rated OCD symptom severity data (CY-BOCS) and pre-treatment depressive symptom severity data (BDI-Y). Owing to variation in the number of CBT sessions received across participants, post-treatment scores were calculated using a last observation carried forward approach. There were no differences in baseline characteristics, including depressive symptom severity, between those in the treatment subsample and full baseline sample (Supplementary material, Table 1).

### Frequency of Depression in Paediatric OCD

**Fig. 1:**
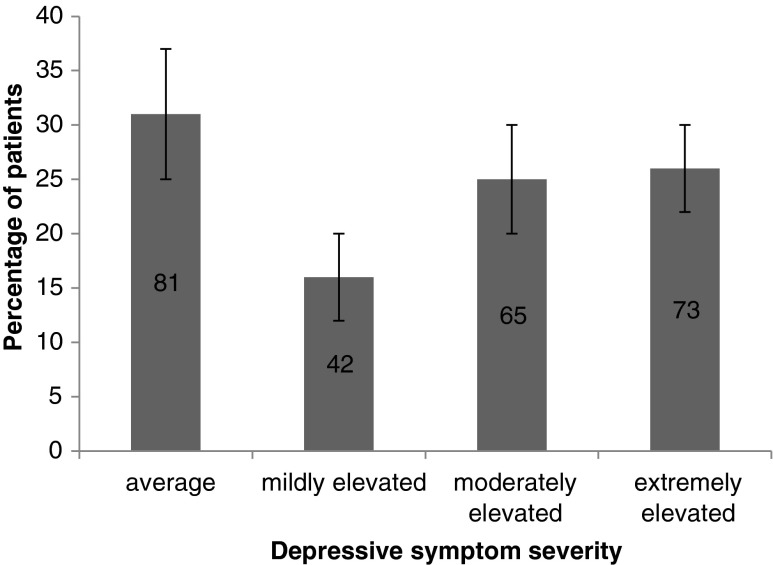
Proportions with 95 % confidence intervals of young people with OCD reporting depressive symptoms

The first aim of the current study was to examine the prevalence of depression in paediatric OCD. Prior to CBT, over 50 % of the baseline sample (*n* = 295) scored in the moderately (25 %) or extremely (26 %) elevated range for self-reported depressive symptoms (Fig. 1) (22 % and 28 % in the treatment subsample [*n* = 100]). Thirty three out of the 127 (26 %; 95 % CI: 18 – 34 %) young people with DAWBA data met criteria for a suspected depressive disorder (16 out of 63 [25 %] in the treatment subsample).

### Clinical and Demographic Correlates of Depression in Paediatric OCD

The second aim of the current study was to explore the demographic and clinical correlates of self-reported depressive symptoms (BDI-Y) and suspected depressive disorders (DAWBA) in the baseline sample (Tables 1 and 2).^1^ More females (48 %) than males (15.3 %) self-reported extremely elevated depressive symptoms; χ^2^ = 28.45, *p* < 0.001. Similarly, more females than males met criteria for a suspected depressive disorder. Neither depressive symptoms nor suspected disorders were associated with age at assessment or of OCD onset. Depressive symptoms and suspected diagnoses were both associated with poorer global functioning and children with a suspected depressive disorder were more likely to have had a psychiatric admission prior to assessment and less likely to be attending school than those without depression. Rates of familial OCD and depression, SSRI medication and prior CBT were comparable between those with and without depression diagnoses.

**Table 1:** Demographic and clinical correlates of depression in children with OCD prior to treatment (*N* = 295)

	Depressive symptoms (*n* = 261)	Suspected depressive disorders (*n* = 127)		
	Pearson’s *r*	Not depressed (*n* = 94)	Depressed (*n* = 33)	Chi square/*t*-test
Gender; female, n (%)	--	58 (40.8)	25 (62.5)	8.55*
Age (years), mean (SD)	0.12	14.57 (2.55)	14.97 (2.34)	−0.79
Age at onset (years), mean (SD)	0.05	10.30 (3.14)	10.53 (3.32)	−0.35
Family history of affective disorder	--	45 (48.9)	11 (34.4)	2.03
Family history of OCD	--	15 (16.3)	6 (18.8)	0.10
Previous CBT	--	58 (41.4)	16 (41.0)	0.01
Current SSRI medication, n (%)	--	50 (36.8)	17 (43.6)	0.60
Global functioning (CGAS), mean (SD)	−0.19*	46.94	38.00	3.89*
Psychiatric admission	--	12 (12.9)	11 (34.4)	7.31*
School non-attendance	--	15 (22.7)	15 (50.0)	7.14*
OCD severity, mean (SD)				
Clinician (CY BOCS)	0.42*	25.62 (6.45)	31.64 (5.18)	−4.84*
Parent (ChOCI)	0.29*	30.69 (8.91)	36.93 (9.26)	−3.16*
Child (ChOCI)	0.48*	28.16 (8.55)	38.71 (6.65)	−5.40*
OCD symptoms dimensions (CY-BOCS), mean (SD)				
Symmetry	0.18*	1.77 (1.49)	1.97 (1.33)	−0.67
Forbidden thoughts	0.34*	4.62 (3.65)	5.74 (4.32)	−1.32
Checking	0.23*	3.80 (3.33)	4.30 (3.45)	−0.73
Hoarding	0.23*	0.68 (0.88)	0.91 (1.01)	−1.31

*CGAS* Children’s global functioning Scale, *CY-BOCS* Children’s Yale-brown obsessive compulsive scale, *ChoCI* Children’s obsessive compulsive inventory *SD* standard deviation, * p < 0.01

**Table 2:** Means (standard deviations) and t-tests for OCD symptom severity, child depression before and after CBT treatment in children with OCD

	Baseline		Post-treatment		t (df)
	n	Mean (sd)	n	Mean (sd)	
OCD severity Clinician (CY-BOCS)	295	27.50 (6.43)	112	14.88 (8.06)	17.98 (111)*
Parent (ChOCI-p)	222	33.62 (9.36)	56	21.04 (11.32)	6.85 (41)*
Child (ChOCI-ch)	216	31.78 (8.66)	53	21.04 (11.32)	9.63 (46)*
Depression (BDI-Y, t-score)	261	62.66 (13.04)	62	53.53 (12.80)	5.90 (58)*
Global functioning (CGAS)	218	45.32 (13.02)	141	74.36 (19.35)	−11.51 (88)*

*p < 0.001

*CY-BOCS* Children’s Yale-brown obsession and compulsion scale, *ChOCI-p/-ch* Children’s Obsessive-compulsive inventory – parent/child version, *BDI-Y* Beck’s Depression inventory for youth, *CGAS* Clinician-rated global assessment scale

Depressive symptoms and suspected disorders were associated with greater pre-treatment OCD severity as rated by all informants. Depressive symptoms were correlated with all OCD dimensions, especially with the Forbidden thoughts dimension; although this was not significantly stronger than with the other dimensions. There were no differences in OCD dimensions between those with and without suspected depressive disorders.

### Depression and CBT Treatment Outcomes

The final aim of the current study was to examine the effect of depressive symptoms and suspected disorders on CBT treatment outcomes. OCD, depressive symptom severity and global functioning all significantly improved from pre- to post-treatment (Table 2). Clinician-rated OCD severity was in the upper end of the moderate range (19 – 30) prior to CBT and lower end of mild range (11 – 18) following treatment (Micali et al. 2010). Depressive symptom severity was moderately elevated (60 – 69) prior to treatment and average or below (<55) after treatment (Beck et al. 2001). Children demonstrated obvious problems (41–50) in global functioning at assessment which decreased to minor impairments (71–80) post-treatment (Shaffer et al. 1983).

**Fig. 2:**
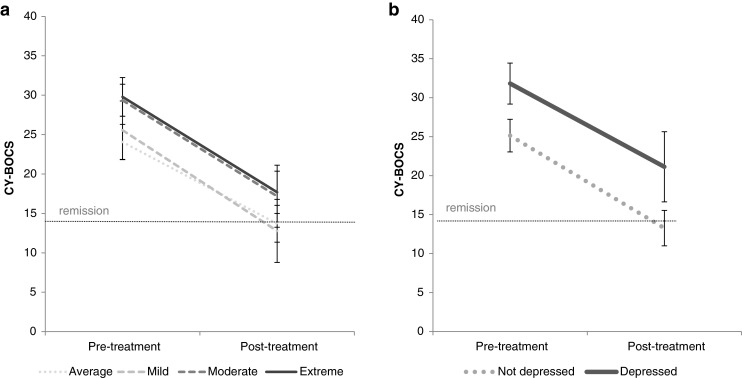
Clinician-rated OCD symptom severity before and after CBT treatment in young people with OCD and concurrent depression (**a** depressive symptoms, **b** depressive disorders)

Mixed-model ANOVAs of OCD symptom severity were conducted with Time (pre- vs. post-treatment) as the within-subjects factor and i) Depressive Symptom Severity (average, mildly, moderately, extremely elevated) or ii) Suspected Depressive Disorder (absent vs. present) as between-subjects factors. Owing to too few patients with parent- and child-reported OCD symptom severity at post-treatment (ns = 56 and 53, respectively), ANOVAs were only conducted for clinician-rated OCD (CY-BOCS) (Fig. 2). For analyses both on the basis of Depressive symptom severity and on the basis of Suspected Depressive Disorders, there were significant main effects of Time on OCD severity, (Symptoms; *F* (1, 96) = 288.551, *p* < 0.001, *η* ^2^ = 0.75: Suspected Depressive Disorder; *F* (1, 61) = 118.83, *p* < 0.001, *η* ^2^ = 0.66), confirming OCD severity improved for all depression groups. There were significant main effects of the groups formed on the basis of Depressive symptom severity (*F* (3, 96) = 4, 37, *p* < 0.01, *η* ^2^ = 0.12) and Suspected Depressive Disorder (*F* (1, 61) = 15.04, *p* < 0.001, *η* ^2^ = 0.20) but crucially there were no significant Time x Depressive Symptom Severity/Disorder interactions (*Fs* < 0.62, *ns, η* ^2^ < 0.02), indicating that youth with depression had more severe OCD both before and after CBT, but improved to an equivalent extent to those with less severe depressive symptoms or without suspected depressive disorders.

The effect of depressive symptom severity and suspected depressive disorders on response and remission rates was also examined (see Table 3 in supplementary material).

Exploratory multiple regression analyses examined the impact of depressive symptoms (Models 1 and 2) and disorders (Models 3 and 4) on post-treatment clinician-rated OCD severity (Table 3). Analyses examined the possible confounding effects of sex, concurrent SSRI medication and pre-treatment OCD severity (in models 2 and 4) in predictions for both depressive symptoms and suspected diagnoses. Sex did not predict post-treatment OCD severity (*βs* < 0.06, *ns*). Interestingly, being on SSRI medication predicted worse post-treatment OCD symptom severity (*βs* > 0.44, *p* < 0.01). Greater depressive symptom severity and meeting criteria for a depressive disorder predicted worse post-treatment OCD severity (*βs* = 0.19 and 0.26, *ps* < 0.05, respectively). However, these became non-significant when pre-treatment OCD severity was included in the models (*βs* = 0.02 and 0.13, *ns*).

**Table 3:** Multiple regression analyses predicting children’s post-treatment OCD symptom severity (clinician-rated) following CBT (total *n* = 100)

	Depressive symptom severity (BDI-Y)				Depressive Disorders (DAWBA			
	Model 1 *n* = 98		Model 2 *n* = 98		Model 3 *n* = 61		Model 4 *n* = 61	
	β	t	β	t	β	t	β	t
Sex	−0.05	−0.48	−0.06	−0.65	0.06	0.56	0.04	0.33
SSRI medication	0.44	4.84^**^	0.28	3.34^**^	0.48	4.32^**^	0.32	2.93^**^
Pre-treatment depression	0.19	1.98^*^	0.02	0.20	0.26	2.14^*^	0.13	1.16
Pre-treatment OCD severity (CY-BOCS)	--	--	0.47	5.29*^**^	--	--	0.41	3.56^**^
R^2^	0.26		0.43		0.37		0.49	
F (df)	11.14 (3,95)^***^		17.71 (4,94)^***^		11.36 (3,58)^**^		13.41 (4,57)^***^	

* *p* < 0.05 ** *p* < 0.01

## Discussion

This study examined the prevalence and clinical presentation of depression in paediatric OCD and its impact on CBT response. To our knowledge, this is the largest study to date in an out-patient setting. It is also the first to examine both dimensional and diagnostic measures of depression and to include multiple informants of OCD severity. Results were largely consistent across the different measures.

As predicted, and consistent with previous paediatric findings (Ivarsson et al. 2008; Storch et al. 2012), clinical levels of depression were common, affecting around a quarter of patients. Taken together with findings from previous studies, this finding indicated the depression is one of the most common comorbidities in paediatric OCD. There are a number of possible reasons for this. Depression may be common because of the distress and functional impairments associated with experiencing OCD. Alternatively, the shared genetic and environmental aetiology of depression and OCD may explain their frequent co-occurrence (Bolhuis et al. 2013). Depression was more common in girls than boys in agreement with previous studies with youth OCD samples (Leonard et al. 2014; Storch et al. 2012). This is in accordance with the female preponderance of depression generally (Nolen-Hoeksema 2001) which may be attributed to sex differences in biological or environmental determinants (Parker and Brotchie 2010).

Depressive symptoms and suspected disorders were associated with greater OCD severity prior to treatment, regardless of OCD informant. Greater depressive symptom severity was associated with more OCD symptoms across all OCD dimensions, especially forbidden thoughts containing aggressive, sexual, religious and somatic obsessions and checking compulsions. This is in agreement with previous studies demonstrating stronger associations between depression and these symptom categories (Fernández de la Cruz et al. 2013; Hasler et al. 2005; Hong et al. 2004), and with studies suggesting these symptoms are the most distressing (Abramowitz et al. 2003). However, there were no differences in OCD dimension scores between those with and without suspected depressive disorders, perhaps because of smaller sample sizes for these comparisons.

Depressive symptoms and depressive disorders were associated with poorer global functioning, in line with limited previous studies (Storch et al. 2012). Risk (e.g., harm to self) is common in depression which would contribute to functional impairments. Additionally, depression is characterised by decreased motivation and withdrawal which may confer poorer functioning in a number of areas. This study was the first to examine specific markers of functional impairment in the context of depression. Children with suspected depressive disorders demonstrated higher rates of psychiatric hospitalisation and school non-attendance. Greater OCD symptom severity and poorer global functioning in youth with depression highlight the clinical significance of this specific comorbidity in paediatric OCD.

Reassuringly, depressive symptoms decreased after treatment in-line with reductions in OCD symptoms. This could reflect the fact that depression results from OCD-related distress, so as OCD symptoms reduce so too does depression. This is in agreement with limited studies indicating that OCD tends to precede depression (Anholt et al. 2011; Diniz et al. 2004) and with a recent multi-modal treatment study showing that average reductions in OCD severity were associated with reductions in depressive symptoms after treatment in youth with OCD (Meyer et al. 2014). However, other studies demonstrate reciprocal longitudinal associations between obsessive-compulsive and depressive symptoms (Bolhuis et al. 2013). Alternatively, elements of CBT for OCD could directly effect depression. For example, graded exposure to fear situations may overlap with behavioural activation; an effective treatment for paediatric depression (Weisz et al. 2006). Further research is needed to examine the mechanisms by which children’s depressive symptoms decrease across treatment.

Youth with depression demonstrated greater OCD symptom severity both before and after CBT. However, depressive symptom severity and suspected depressive disorders did not predict worse post-treatment OCD severity when accounting for baseline OCD severity, suggesting depression is not independently associated with worse outcomes. In other words, individuals with depressive symptoms or disorders showed equivalent improvements in OCD symptoms following CBT, with or without concomitant SSRI medication, as compared to those who were not depressed.

The lack of attenuating effect is in contrast to studies showing greater post-treatment OCD severity in those with comorbid MDD (Abramowitz and Foa 2000; Storch et al. 2008). However, our findings are in line with other studies showing no attenuating effect of depression diagnoses on CBT outcomes (Farrell et al. 2012). This clarifies the mixed results seen in previous studies, suggesting that depression may be a marker for more severe general symptomatology.

Interestingly, being on SSRI medication significantly predicted worse post-treatment OCD. This seems counterintuitive given evidence from randomised controlled trials showing superior outcomes following CBT in combination with SSRI medications compared to CBT alone (Garcia et al. 2010). However, in the current sample medication could represent a proxy for more complex or perhaps treatment-resistant OCD given that SSRI medication was determined by clinical judgement rather than random allocation, as in clinical trials.

The lack of attenuating effect of depression indicates that clinicians should continue to treat paediatric OCD as usual in the presence of depression and depression will also improve across treatment. However, associations between OCD and depressive symptoms and depressive disorders suggest that children with more severe OCD are characterised by a more complex clinical picture, highlighting the need for clinicians to screen for depressive symptomatology.

## Limitations

The strengths of the current study include the multi-method analytic approach and the multiple informants of OCD. However, owing to the naturalistic design of the study there are a number of limitations. First, although CBT was protocol-driven there is likely to be variation in a number of factors. Although the CBT protocol was designed to target OCD symptoms specifically, skilled clinicians may have also addressed depressive symptoms, removing any attenuating effect. Second, depressive disorders were identified using the self-administered DAWBA rather than structured clinical interviews. However, the DAWBA demonstrates substantial inter-rater agreement with clinical interview measures (Aebi et al. 2012), and the frequency of depression diagnoses in the current sample was comparable to those in other studies suggesting that DAWBA diagnoses of depression provided a good proxy for clinical diagnoses. Third, as is often the case with studies of this kind, we cannot be sure whether depressive symptoms were comorbid to OCD, perhaps reflecting a shared vulnerability, or secondary as a result of OCD-related distress. Future research should examine the impact of temporal associations between OCD and depression in young people on treatment response. Finally, we were unable to examine post-treatment outcomes for parent and child ratings of OCD owing to small samples.

## Conclusions

Depression frequently co-occurs with paediatric OCD and is associated with more severe obsessive-compulsive symptomatology and functional impairment. However, the lack of attenuating effect of depression on CBT, with or without SSRI medication, suggests clinicians should continue to treat paediatric OCD as usual in the presence of concurrent depressive symptoms and depression will also improve across treatment.

## Electronic supplementary material

Below is the link to the electronic supplementary material.

ESM 1 (DOCX 14 kb)

ESM 2 (DOCX 12 kb)

ESM 3 (DOCX 16 kb)

## Footnotes

1 Analyses were repeated on the treatment subsample but revealed a similar pattern. The results of the full sample are reported to retain maximum power (data available on request).
