# Supplementary material for: Paediatric Obsessive-Compulsive Disorder and Depressive Symptoms: Clinical Correlates and CBT Treatment Outcomes
Source: J Abnorm Child Psychol. 2014 Oct 10;43(5):933–42. doi: 10.1007/s10802-014-9943-0 (PMC4465665; doi:10.1007/s10802-014-9943-0)
Supplement: Supplementary file 1 — (DOCX 14 kb) [file 10802_2014_9943_MOESM1_ESM.docx]

Table A1. Descriptives of paediatric OCD sample

|  | Total sample  (n = 295) | Assessment-only  (n = 195) | Treatment subsample  (n = 100) | Differences between assessment-only and treatment subsamples,  χ^2^/*t*-test (df), *p* |
| --- | --- | --- | --- | --- |
| Gender; female: male, *n, (%)* | 125:170 (42:58) | 70: 113 (38: 62) | 55:57 (49: 51) | 3.35 (1), *p=* .067 |
| Age [yrs], *M, (SD)* | 14.73 (2.39) | 14.63 (2.37) | 14.89 (2.42) | -.89 (287), *p=* .376 |
| Age at onset [yrs], *M, (SD)* | 10.57 (3.10) | 10.44 (2.96) | 10.78 (3.32) | -.91 (283), *p=* .366 |
| Family history of affective disorder, *n,( %)* | 96 (43) | 42 (42) | 49 (45) | .24, (1) *p=* .622 |
| Family history of OCD, *n (%)* | 36 (12) | 16 (10) | 20 (18) | 4.86 (1), *p=* .027* |
| Previous CBT, *n (%)* | 132 (46) | 81 (47) | 51 (46) | .05 (1),  *p=* .903 |
| Current SSRI medication, *n (%)* | 123 (43) | 83 (47) | 40 (36) | 3.14 (1), *p=* .076 |
| Psychiatric hospital admission, *n (%)* | 37 (17) | 19 (18) | 19 (17) | .11 (1), *p=* .857 |
| School non-attendance, *n (%)* | 43 (28) | 24 (28) | 19 (28) | .01 (1), *p=* .959 |

* *p* < .05
