# Supplementary material for: Paediatric Obsessive-Compulsive Disorder and Depressive Symptoms: Clinical Correlates and CBT Treatment Outcomes
Source: J Abnorm Child Psychol. 2014 Oct 10;43(5):933–42. doi: 10.1007/s10802-014-9943-0 (PMC4465665; doi:10.1007/s10802-014-9943-0)
Supplement: Supplementary file 2 — (DOCX 12 kb) [file 10802_2014_9943_MOESM2_ESM.docx]

**Table A2**. Differences between completers and non-completers of the DAWBA diagnostic measure

|  | Completer  N = 127 | Non-completer  N = 168 | Chi-square/t-test (df), *p*-value |
| --- | --- | --- | --- |
| Gender (Female: Male)  *N (%)* | 57:70 (45:55) | 68:100 (41:60) | .58 (1), *p* = .448 |
| Age (years)  *Mean (SD)* | 14.78 (2.31) | 14.67 (2.49) | .38 (287), *p* = .708 |
| Global functioning (CGAS)  *Mean (SD)* | 44.60 (11.11) | 44.31 (11.71) | -.17, (186), *p* = .863 |
| OCD *Mean (SD)*  Clinician(CY-BOCS)  Child (ChOCI-Ch)  Parent (ChOCI-P) | 27.18 (6.67)  30.66 (9.28)  32.60 (9.41) | 26.34 (5.42)  29.99 (8.64)  33.54 (9.15) | -1.20 (293), *p* = .233  -.56 (220), *p* = .578  .74 (214), *p* = .489 |
| Depressive symptoms(BDI-Y)  *Mean (SD)* | 60.29  (12.50) | 61.99  (13.29) | 1.06 (259), *p* = .291 |

*DAWBA* Development And Wellbeing Assessment; *CY-BOCS* Children’s Yale-Brown Obsessive Compulsive Scale, *ChOCI* Children’s Obsessive Compulsive inventory, *Ch* child self-report, *P* parent-report of child, *BDI-Y* Beck’s Depression Inventory for Youth; *df* degrees of freedom; *p* significance value *SD* standard deviation
