# Supplementary material for: Paediatric Obsessive-Compulsive Disorder and Depressive Symptoms: Clinical Correlates and CBT Treatment Outcomes
Source: J Abnorm Child Psychol. 2014 Oct 10;43(5):933–42. doi: 10.1007/s10802-014-9943-0 (PMC4465665; doi:10.1007/s10802-014-9943-0)
Supplement: Supplementary file 3 — (DOCX 16 kb) [file 10802_2014_9943_MOESM3_ESM.docx]

**Appendix 3**. **Examining the effect of depression on response and remission rates in CBT for paediatric OCD**

Treatment responders and remitters were identified using the CY-BOCS in-line with Storch and colleagues’ criteria ([Storch, Lewin, De Nadai, & Murphy, 2010](#_ENREF_1)). Treatment response was defined as a 25% or more reduction in CY-BOCS severity from pre- to post-treatment. Remission from OCD were identified using post-treatment CY-BOCS scores of less than 14.

In total, 86 of the 112 (77%) children who received CBT for their OCD were considered treatment responders and 59 out of 112 (53%) remitted from their OCD following treatment.

There was no difference in baseline depressive symptom severity (BDI-Y) between children who did (m = 61.56 (13.16) and did not (m = 61.91 (13.58) respond to CBT; *t*(98) = .11, *p* = .91. However, children who remit from OCD had significantly lower baseline depressive symptoms (m = 65.02 (13.55)) and non-remitters (m = 68.29 (12.09)); *t*(98) = 2.59, *p* = .01.

There were no significant differences in response or remission rates between those who did and did not meet criteria for a suspected depressive disorder according to the DAWBA (Table A1). However, sample size for these analyses was small and the difference in remission rates approached significance.

Comparable response rates but suggestive evidence for lower remission rates in those with more elevated depressive symptoms or meeting criteria for a suspected depressive disorder reflect findings from the previous studies (REFs) and our interpretation of the analyses of variance in the current study which suggests that children with elevated depression respond to CBT to the same extent as those with less severe depression but are less likely to reach remission cut-off because they begin with worse OCD symptom severity prior to treatment.

Table A3. Treatment response and remission rates (%) for CBT for paediatric OCD in children with suspected deppressive disorders (DAWBA); n = 63

|  | Depression | No depression | χ^2^(df) |
| --- | --- | --- | --- |
| Response  (*25% CY-BOCS reduction*) | 69 | 77 | .39 (1), *p* = .53 |
| Remission  (*Post-treatment CY-BOCS <14)* | 31 | 57 | 3.28 (1), *p* = .07 |

*DAWBA* Development And Well-being Assessment *CY-BOCS* Children’s Yale-Brown Obsessive Compulsive Scale *p* Significance level

Storch, E. A., Lewin, A. B., De Nadai, A. S., & Murphy, T. K. (2010). Defining treatment response and remission in obsessive-compulsive disorder: A signal detection analysis of the children's Yale-Brown obsessive compulsive scale. *Journal of the American Academy of Child and Adolescent Psychiatry, 49*(7), 708-717.
